# Case Report: Early-onset psychosis as a sentinel manifestation of 3q29 deletion syndrome in an adolescent with neurodevelopmental disorders

**DOI:** 10.3389/frcha.2026.1823061

**Published:** 2026-07-15

**Authors:** W. Vidal, B. Masotto, M. Viñas-Jornet, J. A. Ramos-Quiroga, G. Español-Martín

**Affiliations:** 1 Department of Mental Health, Vall D'Hebron University Hospital, Barcelona, Spain; 2 Medical Genetics Group, Vall D'Hebron Research Institute (VHIR), Barcelona, Spain; 3 Department of Clinical and Molecular Genetics, Vall D'Hebron University Hospital, Barcelona, Spain; 4 Psychiatry, Mental Health and Addictions Group, Vall D'Hebron Research Institute (VHIR), Barcelona, Spain; 5 Biomedical Network Research Centre on Mental Health (CIBERSAM), Instituto de Salud Carlos III (ISCIII), Madrid, Spain

**Keywords:** 3q29 deletion syndrome, ADHD, autism spectrum disorder, case report, early-onset psychosis, intellectual disability, neurodevelopmental disorders

## Abstract

**Background:**

3q29 deletion syndrome is a rare genomic disorder characterized by a broad spectrum of neurodevelopmental and psychiatric manifestations, including developmental delay (DD), intellectual disability (ID), autism spectrum disorder (ASD), attention-deficit/hyperactivity disorder (ADHD), and a markedly increased lifetime risk of psychosis and schizophrenia.

**Case presentation:**

We describe a 17-year-old female adolescent with severe congenital heart disease and longstanding neurodevelopmental impairment who developed early-onset recurrent psychotic symptoms. Chromosomal microarray analysis identified a *de novo* heterozygous 1.6 Mb deletion at the 3q29 locus. The emergence of psychotic symptoms served as the pivotal clinical trigger for genetic investigation in the absence of distinctive dysmorphic findings. Combination therapy with lurasidone and olanzapine led to sustained clinical stabilization over a one-year follow-up period, allowing gradual dose adjustment and functional reintegration through adapted educational and recreational activities.

**Conclusions:**

Early-onset psychotic symptoms in adolescents with neurodevelopmental comorbidities may represent a critical clinical indicator of underlying pathogenic copy number variants, including 3q29 deletion syndrome, even in the absence of highly recognizable dysmorphic features. Increased clinical awareness may facilitate consideration of early genetic testing in this clinical profile to support precision-informed diagnostic evaluation, treatment planning and multidisciplinary management.

## Introduction

The 3q29 deletion syndrome is a rare copy number variant first reported in 2001, with a highly variable clinical presentation (1). This autosomal dominant disorder is typically caused by a *de novo* heterozygous 1.6 Mb deletion at chr3:195,998,129–197,623,129 (hg19) (2, 3). The most frequent manifestations are neurodevelopmental and psychiatric, and include developmental delay (DD), mild-to-moderate intellectual disability (ID), autism spectrum disorder (ASD), attention-deficit/hyperactivity disorder (ADHD), anxiety disorders, executive function deficits and psychosis/schizophrenia (4, 5). Other common features include mood disorders, graphomotor weakness, feeding problems, gastrointestinal disorders, ocular and dental anomalies, congenital heart defects, and posterior fossa malformations (6).

Clinically, the syndrome is characterized by a non-specific phenotype, making clinical diagnosis difficult and necessitating a multidisciplinary approach (7). It is one of the most potent genetic risk factors for schizophrenia, with a > 40-fold increased risk (8). Psychosis occurs in ∼20% of patients, with prodromal symptoms in another 15% (6, 9, 10). Recent studies have further refined this psychiatric profile, highlighting a high burden of psychosis-spectrum symptoms and substantial clinical heterogeneity among carriers (11). Importantly, the onset of psychosis or prodromal symptoms may occur earlier than in the general population (<18 years), and the clinical course follows divergent clinical trajectories (10).

Although numerous candidate genes within the deleted region have been proposed to explain the phenotype, including psychotic symptoms, the precise pathogenic mechanism remains unclear (12). To date, more than 200 affected individuals have been identified worldwide (7, 13). Neurodevelopmental and psychiatric manifestations represent the main cause of their functional disability (14). Despite this, no syndrome-specific treatment exists, and current management of psychosis relies on limited case reports or extrapolation from idiopathic schizophrenia guidelines (8, 15, 16).

In this context, the growing recognition of clinically relevant copy number variants in psychiatric populations has led several professional bodies to recommend increased awareness of genetic testing in mental health settings, particularly in patients presenting with neurodevelopmental disorders, intellectual disability, congenital anomalies, or severe psychiatric illness (17). Here, we present the case of a 17-year-old female adolescent with mild intellectual disability, attention-deficit/hyperactivity disorder, autism spectrum disorder, and severe congenital heart disease who developed early-onset psychosis. The emergence of psychotic symptoms prompted renewed genetic evaluation and ultimately led to the diagnosis of 3q29 deletion syndrome. This case highlights the diagnostic value of early comprehensive genetic evaluation in patients with complex and atypical neuropsychiatric presentations.

## Case description

### Past medical history

The patient is a daughter of non-consanguineous parents from Catalonia, with no known family history of genetic disorders. She was diagnosed with severe congenital mitral stenosis at 2 months, which manifested with signs of heart failure. At 12 months, she required a mitral valve replacement with prosthetic implantation. Her subsequent clinical course included multiple cardiovascular complications, many of them after valvular prosthetic replacements, including an ischemic stroke in the right fronto-cortical territory (age 6), and a complete atrioventricular block, requiring permanent pacemaker (age 9). Additionally, she developed an infective endocarditis due to *Staphylococcus lugdunensis* (age 14)*.* At 15 years, she suffered three cardiopulmonary arrests secondary to sustained ventricular tachycardia. As a consequence, she required orthotopic heart transplantation, which was complicated by pulmonary hypertension, right ventricular dysfunction, and diaphragmatic paralysis. Additional history included recurrent episodes of pneumonia, chronic anemia, and an episode of upper gastrointestinal bleeding at 16 years. She had no known drug allergies or history of substance use.

### Neurodevelopmental history

She was born at term (>37 weeks) by forceps-assisted vaginal delivery, with a birth weight of 3,200 g. Neonatal jaundice occurred but did not require phototherapy. No teratogenic exposures or perinatal complications were reported. Early motor milestones, including head control, sitting, and independent walking, were achieved within expected timeframes. However, delayed language acquisition, learning difficulties, and limited peer socialization were observed. Her behavioral profile was characterized by cognitive rigidity, obsessive-compulsive traits, restricted interests, and imaginary friends. Due to the complexity of multiple physical health issues during infancy, a more in-depth investigation was attempted, including karyotyping, which showed no significant findings; nevertheless, a comprehensive evaluation of developmental delay and behavioral features was not completed at that time. Subsequently, she transitioned from mainstream schooling to individualized home-based education at age 11.

### Clinical course

At 17 years, she was referred for a psychiatric evaluation due to longstanding behavioral disturbances including motor restlessness, inattention, impulsivity, poor organization, social withdrawal, and learning difficulties. A comprehensive assessment was conducted using multiple instruments, including the Wechsler Intelligence Scale for Children–Fifth Edition (WISC-V), Autism Diagnostic Observation Schedule–Second Edition (ADOS-2), Kiddie Schedule for Affective Disorders and Schizophrenia for School-Age Children–Present and Lifetime Version DSM-5 (K-SADS-PL-5), Vineland Adaptive Behavior Scales–Third Edition (Vineland-3), and the Conners Rating Scale–Fourth Edition (Conners 4). Vineland-3 results showed significant adaptive functioning impairment (Communication = 68, Daily Living Skills = 43, Socialization = 74; Adaptive Behavior Composite = 62). Findings across these instruments supported the presence of neurodevelopmental disorders, while the clinical interview did not identify an alternative primary psychiatric disorder at that time. Based on the integration of clinical observations, adaptive functioning measures, and neuropsychological assessment, the patient was diagnosed with mild ID, ASD, and ADHD. Following this assessment, a close clinical follow-up plan was established prior to initiating pharmacological treatment. However, two weeks after the initial psychiatric evaluation, the patient developed psychotic symptoms characterized by second-person auditory hallucinations, persecutory delusions, soliloquies, and inappropriate laughter. She subsequently presented to the emergency department, where inpatient admission was deemed necessary for further evaluation.

### Timeline

Table 1

**Table 1:** Timeline of relevant clinical and therapeutic events in the patient's medical course.

Age	Clinical event
Birth	Term delivery, neonatal jaundice without phototherapy
2 months	Diagnosis of severe congenital mitral stenosis
12 months	Mitral valve replacement surgery
6 years	Ischemic stroke in right fronto-cortical territory
9 years	Complete atrioventricular block requiring pacemaker
14 years	Infective endocarditis (*Staphylococcus lugdunensis*)
15 years	Three cardiopulmonary arrests → Heart transplantation
17 years	Psychiatric assessment → Mild ID, ASD and ADHD diagnosis
	^a^Onset of psychotic symptoms → Hospitalization
	Array CGH → Diagnosis of 3q29 deletion syndrome
17–18 years	Recurrence of psychotic symptoms → Initiation of combination therapy with lurasidone and olanzapine
Follow-up	One-year clinical stabilization

a 2 weeks after psychiatric assessment.

### Diagnostic assessment

During hospitalization, pediatric, neurology, and cardiology consultations were performed, and no organic etiology for the psychiatric symptoms was identified. An expanded diagnostic workup was undertaken, including genetic evaluation. Chromosomal microarray analysis (array CGH) was performed on the patient's DNA using the CytoSure Constitutional v3 8 × 60 K oligonucleotide array (OGT), following the manufacturer's protocol. The array features a backbone resolution of approximately 663 kb, enhanced to 189–375 kb within ISCA-defined regions (International Standard Cytogenomic Array Consortium), with exon-level resolution in 354 genes and gene-level resolution for 288 genes curated in the ClinGen Dosage Sensitivity Map. Image acquisition was followed by quantification and analysis using Agilent CytoGenomics software, utilizing the default ADM-2 statistical algorithm with a sensitivity threshold set at 6.1 (Agilent Technologies). Detected copy number variants (CNVs) were aligned to the human reference genome (GRCh37/hg19) and classified according to the American College of Medical Genetics (ACMG) and the Clinical Genome Resource (ClinGen) guidelines (18). This analysis identified a heterozygous interstitial 1.6 Mb deletion at 3q29, consistent with the recurrent deletion associated with 3q29 deletion syndrome (OMIM #609425). Parental studies using array CGH showed that neither parent carried the identified copy number variant. However, original parental laboratory reports were not accessible for inclusion in this manuscript. In addition, a detailed physical examination to assess dysmorphic features revealed a weight of 45.6 kg (8th percentile, −1.41 SD), height 154 cm (5th percentile, −1.67 SD), BMI 19.23 kg/m^2^ (23rd percentile, −0.75 SD), body surface area 1.4 m^2^, and head circumference 53.3 cm (6th percentile, −1.6 SD). The patient exhibited generalized cutaneous and mucosal pallor, with thick, normally implanted hair. Craniofacial evaluation revealed facial asymmetry with right malar prominence, dysmorphic ears with folded helix (5 cm in length), mild downward slanting of the palpebral fissures (length 2.8 cm), decreased interpupillary distance (5 cm), and arched eyebrows. Additional findings included bilateral fifth toe clinodactyly and abnormal “hockey stick” palmar creases.

### Therapeutic intervention

Risperidone was initiated and titrated from 0.5 mg/day to 3 mg/day, with a good clinical response but excessive daytime sedation; finally, she was ultimately discharged on 2.5 mg/day. Following discharge, somnolence persisted, prompting a reduction of risperidone to 1.5 mg/day; however, psychotic symptoms recurred, requiring dose re-escalation to 2.75 mg/day. During this period, she developed fever and malaise and was diagnosed with SARS-CoV-2 pneumonia, requiring another hospitalization. Psychiatric symptoms persisted, leading to switching to lurasidone (up to 37 mg/day).

### Follow-up and outcomes

After discharge, psychotic symptoms worsened; therefore, combination therapy was initiated. First, lurasidone plus risperidone and subsequently lurasidone plus olanzapine. Doses were increased to lurasidone 111 mg/day and olanzapine 10 mg/day, resulting in gradual improvement. Over one year, combination therapy led to progressive clinical stabilization, with marked reduction of psychotic symptoms, improved behavioral regulation, and functional recovery allowing participation in adapted educational and recreational activities. Treatment adherence remained adequate during follow-up. Excessive daytime sedation represented the main adverse effect during risperidone treatment and contributed to subsequent medication adjustments. Currently, the patient remains symptom-free on treatment with lurasidone 74 mg/day, with occasional olanzapine 5 mg as needed for increased anxiety or agitation.

## Discussion

This case contributes to the growing clinical literature describing neuropsychiatric manifestations associated with 3q29 deletion syndrome, highlighting early-onset psychosis as a potential sentinel manifestation within the heterogeneous phenotypic spectrum of this genomic disorder. Our patient had severe congenital heart disease, resulting in multiple medical complications throughout her life that significantly limited her functional development and delayed a comprehensive neurodevelopmental evaluation. In 3q29 deletion syndrome, cardiovascular and gastrointestinal malformations are among the most frequently reported congenital anomalies and are often accompanied by failure to thrive and dental abnormalities. In one of the largest reported cohorts (*N* = 44), cardiac defects were identified in 26% of affected individuals, with patent ductus arteriosus being the most common lesion (2, 3).

The 3q29 locus represents a recurrent subtelomeric, dosage-sensitive genomic region in which both deletions and duplications have been associated with distinct but overlapping clinical phenotypes. Accordingly, two reciprocal genomic disorders have been described: the 3q29 microdeletion syndrome and the 3q29 microduplication syndrome (1, 19). This region spans approximately 1.3–1.6 Mb and encompasses more than 20 protein-coding genes, several of which are involved in synaptic transmission, neuronal signaling, and neurodevelopmental processes. Altered gene dosage within this interval is therefore thought to contribute to the neurological and psychiatric manifestations observed in individuals with either deletions or duplications of 3q29 (1, 19).

In particular, the 3q29 microdeletion has been associated with a broad spectrum of neuropsychiatric and cognitive features, including mild to moderate ID, ASD, and an increased risk for psychiatric disorders. Although no single pathogenic gene has been definitively implicated, the underlying mechanism is likely driven by haploinsufficiency and disruption of gene regulatory networks critical for normal neuronal function (1, 12). The clinical spectrum of 3q29 deletion syndrome is highly heterogeneous, ranging from mild to severe presentations and typically lacking distinctive facial features. This variability often hampers early clinical recognition, particularly in childhood, as occurred in our patient, who exhibited no specific dysmorphic features. Nonetheless, neurocognitive impairment is almost universally reported, with ID and psychiatric disorders representing the most consistent findings (2, 6, 7). In line with previous reports, our patient was diagnosed during adolescence with mild ID, ASD, and ADHD, all of which had been evident since early childhood.

Although this neurodevelopmental profile is not uncommon in pediatric neurology practice and may overlap with multiple genetic conditions, the emergence of early-onset recurrent psychosis that was only partially responsive to monotherapy served as a pivotal clinical indicator prompting genetic investigation (6). This observation underscores the potential role of early-onset psychosis as a clinical sentinel marker of underlying copy number variants in adolescents with complex neurodevelopmental comorbidities. Recent studies have emphasized that psychosis-spectrum symptoms are a prominent and clinically relevant component of 3q29 deletion syndrome, often emerging early and following heterogeneous clinical trajectories (11).

While the recurrent 3q29 deletion identified in our patient provided a molecular diagnosis consistent with the observed phenotype, substantial clinical heterogeneity has been reported among affected individuals. This variability suggests that additional genetic or environmental modifiers may influence phenotypic expression. In selected cases with atypical manifestations or unexplained clinical severity, further genomic investigations such as whole-exome sequencing may provide complementary information and improve understanding of genotype–phenotype relationships (17).

Pharmacological management of psychosis in the context of pathogenic copy number variants can be particularly challenging. In our patient, risperidone initially achieved partial symptom control but was limited by sedation, with symptom recurrence upon dose reduction. Subsequent incomplete responses to sequential monotherapy trials required combination treatment with lurasidone and olanzapine. Notably, the sustained stabilization achieved with combination therapy in our patient contributes additional longitudinal data to the limited literature describing pharmacological outcomes in this population. This treatment trajectory is consistent with emerging evidence suggesting variable antipsychotic responsiveness and a potential vulnerability to treatment resistance in individuals with 3q29-associated psychosis (6, 9, 16).

Although clozapine remains the treatment of choice for treatment-resistant schizophrenia and has been reported as an effective option in some individuals with 3q29 deletion syndrome-associated psychosis (14, 20, 21), escalation to clozapine was not considered necessary in the present case because sustained clinical stabilization was achieved with lurasidone–olanzapine combination therapy. The decision to switch to lurasidone was supported by evidence demonstrating its efficacy and safety profile in adolescents with schizophrenia, including randomized controlled trials and subsequent comparative analyses showing a favorable efficacy profile together with relatively limited effects on body weight, metabolic parameters, prolactin levels, QT interval, and overall cardiovascular safety compared with several other second-generation antipsychotics (22–24). These considerations were particularly relevant given the patient's history of severe congenital heart disease, previous ventricular arrhythmias, orthotopic heart transplantation, and excessive daytime sedation during previous risperidone treatment. Although olanzapine was subsequently added because of persistent psychotic symptoms and incomplete response to sequential monotherapy trials, the combination ultimately resulted in sustained symptom remission, improved behavioral regulation, and functional recovery throughout follow-up. Given the favorable clinical response obtained, the overall risk–benefit balance did not support escalation to clozapine.

Overall, this case illustrates the complex, multisystemic phenotype of 3q29 deletion syndrome and emphasizes the necessity of coordinated, multidisciplinary care. Early genetic diagnosis facilitates tailored medical surveillance, anticipatory guidance, and individualized treatment planning, which are essential for optimizing outcomes and improving quality of life in this vulnerable patient population (6, 7, 9). Compared with previously published reports of 3q29 deletion syndrome, our case provides several clinically relevant contributions. While earlier reports have primarily focused on neurodevelopmental phenotypes, dysmorphic features, or severe treatment-resistant psychosis requiring clozapine, psychotic symptoms in our patient represented the principal clinical trigger for a comprehensive genetic re-evaluation during adolescence, ultimately leading to the diagnosis of 3q29 deletion syndrome (4, 13, 14).

In addition, this report offers detailed longitudinal information regarding pharmacological management, documenting sustained stabilization with lurasidone–olanzapine combination therapy following incomplete responses and tolerability limitations associated with antipsychotic monotherapy. The case also illustrates the diagnostic challenges posed by a highly complex multisystem presentation, including severe congenital heart disease requiring heart transplantation. Given the limited literature specifically addressing longitudinal psychiatric management in 3q29-associated psychosis, this case may provide clinically relevant information for psychiatrists and multidisciplinary teams managing similar patients.

### Limitations

This study has several limitations that should be acknowledged. First, as a single case report of 3q29 deletion syndrome, the findings cannot be generalized to the broader population, and causal inferences regarding genotype–phenotype relationships cannot be established. Second, although parental genetic testing was reported as negative for the identified copy number variant, original laboratory reports were not available for independent verification. Third, the assessment of long-term treatment response was based primarily on clinical evaluation and longitudinal follow-up rather than standardized psychometric instruments such as the PANSS. Finally, the absence of comprehensive neurobiological or functional imaging data limits deeper characterization of the pathophysiological mechanisms underlying the psychiatric presentation.

## Conclusions

This case highlights the importance of considering 3q29 deletion syndrome in patients presenting with complex neurodevelopmental profiles, especially when early-onset psychosis or treatment-resistant psychiatric symptoms are present, even in the absence of dysmorphic features. Greater awareness of pathogenic copy number variants among both child and adolescent psychiatrists and adult psychiatrists may facilitate earlier recognition of underlying genetic conditions, reduce diagnostic delays, and support precision-informed psychiatric care. However, findings from individual cases cannot be generalized, and further research is needed to better define genotype–phenotype correlations and optimal management strategies.

## Patient perspective

The patient's family reported that obtaining a unifying genetic diagnosis provided a clearer understanding of the patient's developmental and psychiatric difficulties. They expressed relief at having an explanation that integrated her complex medical and neurodevelopmental history. They also perceived a substantial improvement in daily functioning after stabilization of psychotic symptoms and valued the multidisciplinary follow-up approach. The family hopes that sharing this experience may help clinicians recognize similar cases earlier.

## Data Availability

The original contributions presented in this study are not publicly available because they contain potentially identifiable clinical information. Requests to access de-identified data should be directed to the corresponding author and will be considered in accordance with institutional policies and applicable ethical and privacy regulations.
